# CXADR‐like membrane protein protects against heart injury by preventing excessive pyroptosis after myocardial infarction

**DOI:** 10.1111/jcmm.15955

**Published:** 2020-10-21

**Authors:** Xinglong Han, Zhen‐Ao Zhao, Shiping Yan, Wei Lei, Hongchun Wu, Xing‐Ai Lu, Yueqiu Chen, Jingjing Li, Yaning Wang, Miao Yu, Yongming Wang, Yufang Zheng, Hongyan Wang, Zhenya Shen, Shijun Hu

**Affiliations:** ^1^ Department of Cardiovascular Surgery of the First Affiliated Hospital & Institute for Cardiovascular Science State Key Laboratory of Radiation Medicine and Protection Medical College Soochow University Suzhou China; ^2^ Institute of Microcirculation & Department of Pathophysiology of Basic Medical College Hebei North University Zhangjiakou China; ^3^ Hebei Key Laboratory of Critical Disease Mechanism and Intervention Zhangjiakou China; ^4^ MOE Key Laboratory of Contemporary Anthropology at School of Life Sciences and Zhongshan Hospital Fudan University Shanghai China; ^5^ Obstetrics and Gynecology Hospital State Key Laboratory of Genetic Engineering at School of Life Sciences Institute of Reproduction and Development Fudan University Shanghai China; ^6^ Institute of Developmental Biology & Molecular Medicine Fudan University Shanghai China; ^7^ Key Laboratory of Reproduction Regulation of NPFPC Collaborative Innovation Center of Genetics and Development Fudan University Shanghai China; ^8^ Children’s Hospital of Fudan University Shanghai China

**Keywords:** CLMP, fibroblast, inflammation, myocardial infarction, pyroptosis

## Abstract

Myocardial infarction (MI) results in cardiomyocyte death and ultimately leads to heart failure. Pyroptosis is a type of the inflammatory programmed cell death that has been found in various diseased tissues. However, the role of pyroptosis in MI heart remains unknown. Here, we showed that CXADR‐like membrane protein (CLMP) was involved in pyroptosis in the mouse MI heart. Our data showed that CLMP was strongly expressed in fibroblasts of the infarcted mouse hearts. The *Clmp* ^+/−^ mice showed more serious myocardial fibrosis and ventricular dysfunction post‐MI than wild‐type (*Clmp* ^+/+^) mice, indicating a protective effect of the fibroblast‐expressed CLMP against MI‐induced heart damage. Transcriptome analyses by RNA sequencing indicated that *Il‐1β* mRNA was significantly increased in the MI heart of *Clmp* ^+/−^ mouse, which indicated a more serious inflammatory response. Meanwhile, cleaved caspase‐1 and Gasdermin D were significantly increased in the *Clmp* ^+/−^ MI heart, which demonstrated enhanced pyroptosis in the *Clmp* knockdown heart. Further analysis revealed that the pyroptosis mainly occurred in cardiac fibroblasts (CFs). Compared to wild‐type fibroblasts, *Clmp* ^+/−^ CFs showed more serious pyroptosis and inflammatory after LPS plus nigericin treatment. Collectively, our results indicate that CLMP participates in the pyroptotic and inflammatory response of CFs in MI heart. We have provided a novel pyroptotic insight into the ischaemic heart, which might hold substantial potential for the treatment of MI.

## 1 INTRODUCTION

According to recent reports, approximately 7.9 million (3% of the total population) individuals suffer from myocardial infarction (MI) in the United States, which accounts for approximately 14% of mortality.^1^ Cardiomyocytes die within minutes after coronary artery occlusion in the non‐perfused territory. However, the endogenous regenerative capacity of the heart appears insufficient to compensate for the large loss of functional cardiomyocytes. The loss of cardiomyocytes and scar formation lead to chamber dilatation, contractile dysfunction and, ultimately, heart failure. The prevention of cardiomyocyte death at the early stage of MI represents a promising strategy to reduce the infarct size.^2^ However, there is still no effective therapy for preventing MI‐induced cell death.

To date, two important forms of programmed cell death (PCD), including apoptosis and programmed necrosis, have been demonstrated to be involved in MI. Apoptotic caspases, such as caspase‐3, are activated during ischaemia‐induced cardiac apoptosis, which is a non‐inflammatory form of PCD.^3^ The receptor‐interacting protein kinase 3 (RIP3) and calmodulin‐dependent protein kinase II (CaMKII) represent critical regulators of programmed necrosis, an inflammatory form of PCD, and mediate adverse remodelling after MI.^4^, ^5^ Pyroptosis is another type of inflammatory PCD triggered by caspase‐1 or caspase‐11 (caspase‐4/‐5 in humans).^6^ In the canonical pathway, caspase‐1 plays a crucial role in the formation of inflammasome and cytokine maturation. Active caspase‐1 directly cleaves the precursor cytokines pro‐IL‐1β and pro‐IL‐18 into their mature forms, which further lead to inflammation, vasodilation and immune cell extravasation.^7^ In addition, Gasdermin D (GSDMD) is a substrate of active caspase‐1, and acts as the direct and final executor of pyroptosis.^8^, ^9^ It is well known that a complex inflammatory phase exists at the early stage of MI. However, few papers have reported whether pyroptosis participates in this process.

Cardiac fibroblasts (CFs), a large population of the non‐myocytes in myocardial tissue, are responsible for the homoeostasis of the extracellular matrix (ECM) in the heart.^10^ After acute MI, CFs can transform into cardiac myofibroblasts and secret ECM, which maintains the structural integrity of the left ventricle (LV).^11^, ^12^, ^13^ Moreover, increasing evidences suggest that CFs exhibit a remarkable functional pluralism, including inflammation, proliferation, adhesion and apoptosis.^14^, ^15^, ^16^ The loss of CF‐specific GRK2 leads to decreased secretion of pro‐inflammatory cytokines and decreased infiltration of neutrophils to the ischaemic region.^17^ In particular, myocardial ischaemia/reperfusion (I/R) injury mainly stimulates inflammasome activation in mouse CFs, but not in cardiomyocytes.^18^ These results indicate CFs play important roles during MI.

CXADR‐like membrane protein (CLMP), a member of the CTX family, is a type I transmembrane protein, including an extracellular region, a transmembrane region and a cytoplasmic tail. The amino acid sequences are highly homologous between mice and humans (93%).^19^, ^20^ Previous studies have identified CLMP as a tight junction protein or adhesion junction protein involved in adipocyte maturation and the development of obesity, germ cell translocation, small intestine development and so on.^21^, ^22^ Despite its abundant expression in heart tissues, little is known about the biological functions of CLMP in heart development and the pathological process of cardiac diseases.^23^

Here, we found that CLMP was highly up‐regulated in CFs of the infarcted zone after MI. Moreover, the heterozygous mice showed increased infarct area and deteriorated cardiac function compared with wild‐type mice in MI model. Further studies indicated that the MI‐elevated CLMP might serve as a brake on excessive pyroptotic and inflammatory response of CFs, and thus prevent the MI heart from serious myocardial fibrosis and ventricular dysfunction.

## 2 MATERIALS AND METHODS

### 2.1 Mouse maintenance and generation of *Clmp* mutant mice

All experimental protocols involving animals (FVB/NJ mice) in this study were approved by the Laboratory Animal Research Committee of Soochow University. A single copy insertion of the Act‐RFP‐polyA cassette was introduced into mouse DNA by injecting the piggyBac (PB) transposon system into the zygote. Transposase‐free progeny carrying a Act‐RFP‐polyA cassette in the first intron of *Clmp* gene was identified and bred for subsequent experiments.^24^ The polyA signal in the first intron is expected to prematurely terminate *Clmp* transcription and interfere with its expression levels. The genotype was identified by PCR amplification using genomic DNA isolated from the mouse tail, and the test was repeated for three times. Forward primer 1 (F1: CGATAGGAAGCGGGGTTTG) and reverse primer 1 (R1: CCAAGCGGCGACTGAGATG) were used to detect the wild‐type allele (222 bp), while forward primer 2 (F2: CGGGCAACTCAGACACTTA) and reverse primer 2 (R2: GGAGTCCAATGAGACCCAAG) were used to detect the insertional allele (341 bp). *Clmp* mutant mice could also be identified through RFP imaging under UV irradiation. All mice were maintained in a 12/12‐hour light/dark cycle at 23 ± 2°C in a specific pathogen‐free barrier facility. Mice were free to drink and eat.

### 2.2 Mouse myocardial infarction model

Myocardial infarction was induced through ligation of the mid‐left anterior descending artery (LAD) by an experienced microsurgeon (J. L.) blinded to the group designation.^25^ Female mice were used in this study due to the well tolerance and low morbidity.^26^, ^27^ All the mice were randomly divided into three groups (wild‐type‐Sham group, wild‐type‐MI group and *Clmp* ^+/−^‐MI group, n = 15 per group). As previously described, female mice (8 weeks old) were anaesthetized, and then placed on the operating table in the supine position. Nylon sutures were used to permanently occlude the left anterior descending artery.^28^ After closure of the chest wall, mice were placed in a recovery cage at 37°C overnight and then housed normally. No acute mortality of mice was observed during the experimental period. MI was confirmed by myocardial blanching and electrocardiographic changing. Echocardiography was performed before and after LAD ligation.^29^ For echocardiography, the mice were anaesthetized using 2% isoflurane using during the procedure. The Visual Sonics Vevo2100 system equipped with a medium frequency (30 MHz) MS‐400 transducer was operated by an investigator (Z‐A. Z.) blinded to the group designation. Transthoracic echocardiographic analysis was performed with a 12‐mHz probe on mice at day 0 to 28 post‐MI. Analysis of the M‐mode images was performed using Vevo2100 software to measure the left ventricular ejection fraction (LVEF), LV fraction of shortening (LVFS), LV end‐systolic interior diameter (LVID.s), end‐diastolic interior diameter (LVID.d), left ventricular volume at end systole (LV.vol.s) and left ventricular volume at end diastole (LV.vol.d). M‐mode measurements were obtained from at least three beats and then averaged. After 4 weeks, all animals were sacrificed under anaesthesia and the heart samples were harvested for analysis.

### 2.3 Inflammasome stimulation experiments

For the inflammasome stimulation experiments, cardiac myocytes or CFs were plated at a density of 300 000 cells per 35‐mm dish for 24 hours before drug treatment. The cells were primed with 10 μg/mL LPS (Sigma, Cat. No. L2630) for 24 hours, and aggregation of the inflammasome was subsequently induced by 20 μmol/L Nigericin (InvivoGen, Cat. No. tlrl‐nig) for 1 hour. Supernatants and cell lysate were collected for further study.^30^ For the oestrogen treatment, primary CFs were incubated at 300 pg/mL oestrogen (MCE, Cat. No. HY‐B0234) for 48 hours and then underwent the processes of pyroptosis induction. Cell lysate was collected for *Clmp* expression analysis.

### 2.4 LDH cytotoxicity assay

Pyroptosis was quantitated by measuring lactate dehydrogenase (LDH) using a CytoTox 96^®^ Non‐Radioactive Cytotoxicity Assay (Promega, Cat. No. g1780). Blood samples were centrifuged at 1000 *g* for 20 minutes, and the serum was collected for further tests after dilution (1:20) with PBS. Cell culture supernatants were collected and centrifuged for 3 minutes at 200 *g* after drug treatments. According to the instructions, 50 μL serum or cell culture supernatants were transferred to a 96‐well plate and an equal volume of CytoTox 96^®^ Reagent was added to each well. After incubation for 30 minutes, the reaction was stopped by addition of the Stop Solution, and the absorbance signal at 490 nm was recorded. The LDH activity in the cell culture supernatant was presented as a percentage of the total LDH (cell lysate plus culture supernatant).

### 2.5 ELISA analysis

A commercial kit (Abcam, ab197742) was used for the quantitative measurement of IL‐1β protein in mouse myocardial tissue extracts. Briefly, 50 µL standard or sample was added into appropriate wells. And, 50 µL antibody cocktail was then immediately added to all wells. The microplates were incubated on a plate shaker set to 400 rpm for 1 hour under room temperature condition. After washing each well with 3 × 350 µL wash buffer, 100 μL TMB development solution and 100 μL stop solution were added into each well, and OD value at 450 nm was recorded. The collected data were analysed as described.

### 2.6 Statistical analysis

Comparisons between two groups were analysed using Student's *t* test. Comparisons of multiple groups were analysed with one‐way analysis of variance (ANOVA) or two‐way repeated‐measures analysis of variance with the Bonferroni post hoc test. Statistical significance was denoted by a *P*‐value of <.05. All data were presented as the mean ± SEM. All experimental assays were performed at least three times. For sample size calculation, echocardiographic analysis was performed on mice at 14 days post‐MI and LVEF was a reliable parameter. Our previous research showed that the LVEF value was >70% before MI and dropped to ~30% after that.^26^, ^31^ The number (n = 15) was larger enough at >80% power estimated by Clin Calc: http://clincalc.com/stats/samplesize.aspx.

## 3 RESULTS

### 3.1 Enriched expression of *Clmp* in the infarct zone of mouse ischaemic hearts

**FIGURE 1:**
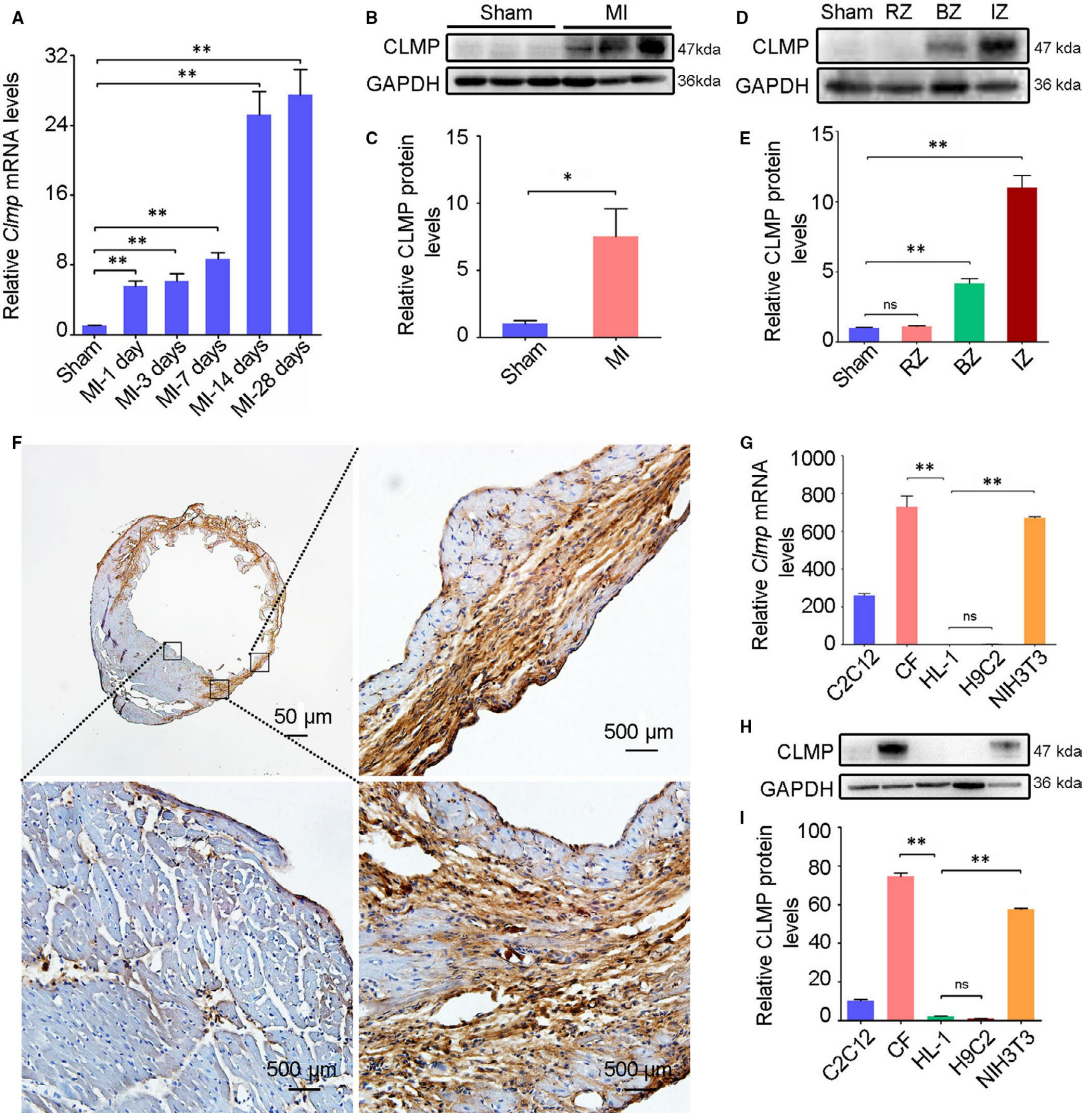
*Clmp* is highly expressed in cardiac fibroblasts and induced in the ischaemic heart. A, The mRNA expression of *Clmp* was significantly increased in cardiac tissue of the wild‐type mice on the indicated days post‐MI (n = 3). B, The representative Western blot imaging of CLMP protein in the sham‐operated and infarcted heart tissues (week 2 after MI). C, The relative densitometric quantification of panel B showed higher expression level of CLMP protein in the ischaemic heart (n = 3). D, The representative Western blot imaging of CLMP protein in the heart lysates from the sham, as well as the non‐ischaemia zone, border zone and infarct zone of the MI heart. E, The relative densitometric quantification of panel D showed the highest expression level of CLMP protein in the IZ of the MI heart(n = 3). F, The representative immunohistochemistry imaging showed significantly increased expression of CLMP in the MI heart. Scale bar indicates 50 μm. G, qRT‐PCR analysis showed the *Clmp* expression in different fibroblasts (CFs: cardiac fibroblasts; NIH3T3) and myogenic cells (HL‐1, C2C12, H9C2). H, Western blot showed abundant expression of CLMP protein in the fibroblasts rather than myogenic cells. I, The relative densitometric quantification of CLMP protein in panel H. All data are presented as the mean ± SEM. Student's t test or one‐way ANOVA; *P < .05; **P < .01; ns, not significant

In order to screen the functional genes in MI, we compared the expression pattern between infarct zone and sham LV using microarray analysis. Among the differentially expressed genes, *Clmp* mRNA expression was enhanced significantly in the infarct zone (data not shown). We then investigated the expression pattern of *Clmp* in healthy and ischaemic mouse hearts. The quantitative real‐time PCR (qRT‐PCR) analysis showed that *Clmp* mRNA was enriched in multiple adult mouse tissues, including the heart (Figure S1), and the *Clmp* mRNA expression was significantly increased in the mouse LV on the indicated days post‐MI compared with the sham control (Figure 1A). Consistent with the qRT‐PCR results, the Western blot analysis showed that CLMP protein was also significantly up‐regulated at approximately 7.5‐fold in the MI heart at week 2 (Figure 1B,C). To determine the localization of CLMP in the MI heart, the tissue lysates from the sham heart, as well as the remote zone (RZ), border zone (BZ) and infarct zone (IZ) of the MI heart, were harvested for Western blot analysis. CLMP was significantly increased in the BZ and IZ of the MI heart, with the highest level in the IZ (Figure 1D,E). Furthermore, the immunostaining showed that CLMP was mainly localized in fibroblasts of the MI heart (Figure 1F). Western blot and qRT‐PCR analyses also demonstrated an abundant CLMP expression in fibroblasts (CFs and NIH3T3) rather than in myogenic cells (C2C12, H9C2 and HL‐1) (Figure 1G‐I). Taken together, these results indicate that CLMP is strongly expressed in the fibroblasts of the ischaemic heart, which suggests that it might play an important role in MI.

### 3.2 Generation and characterization of *Clmp* target mutant mice

**FIGURE 2:**
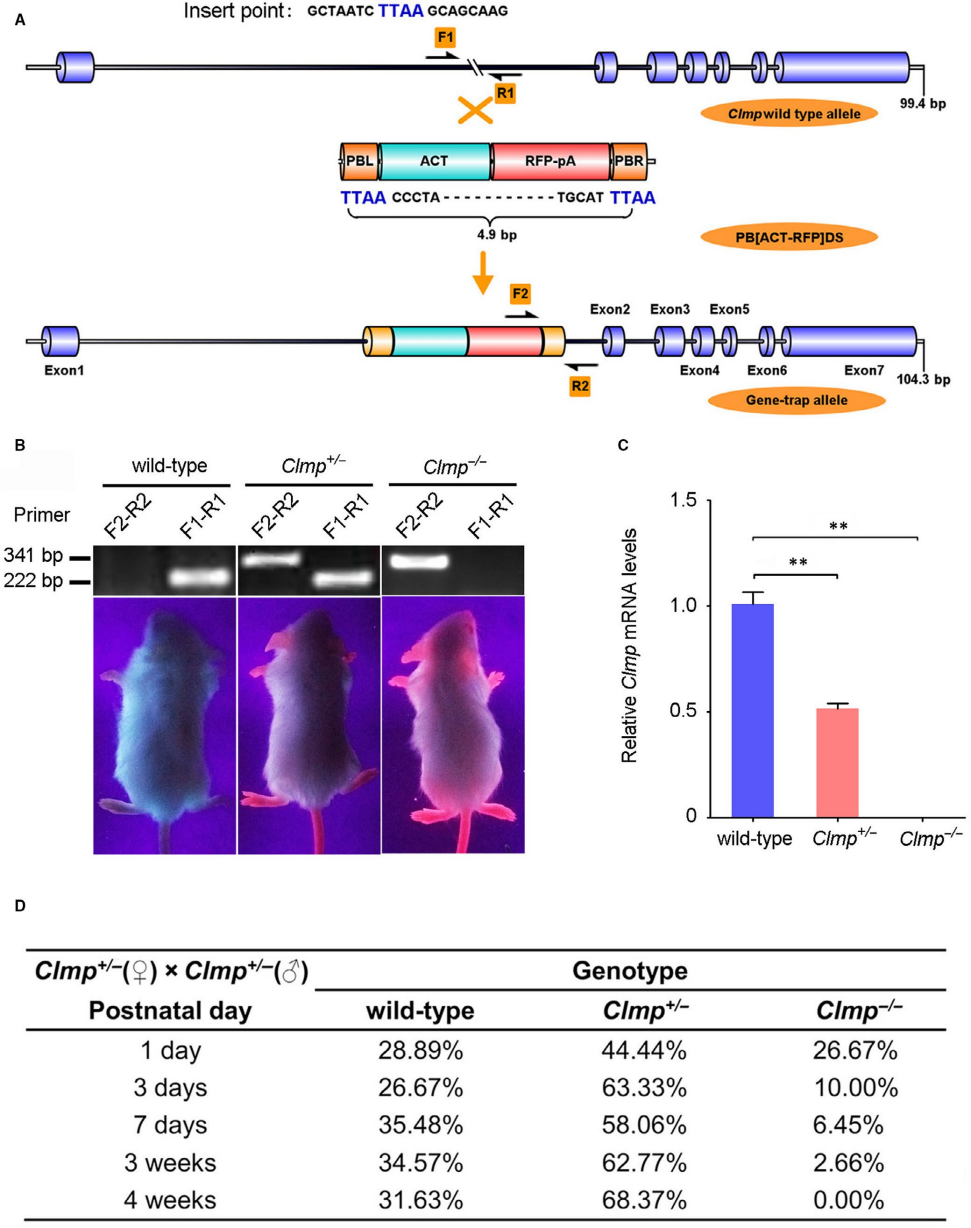
Verification of the piggyBac (PB) transposon insertional mutation. A, The schematic diagram of *Clmp* gene inserted with the PB element. The PB [Act‐RFP, 4.9 bp] element was mapped in the first intron of *Clmp*. F1 and R1 for wild‐type allele (222 bp); F2 and R2 for gene insertional allele (341 bp). B, The representative PCR genotyping and photographs (red fluorescence) of different genotypes (wild‐type, *Clmp* ^+/−^ and *Clmp* ^−/−^). (C) PCR verification of *Clmp* mRNA in the wild‐type, *Clmp* ^+/−^ and *Clmp* ^−/−^ hearts. D, The genetic analysis showed that proportion of neonatal mutant mice gradually deviated from Mendelian Laws due to homozygous lethal (n = 311). All data are presented as the mean ± SEM. One‐way ANOVA; **P < .01

To investigate the function of *Clmp* in MI, we used *Clmp* mutant mice established by the piggyBac transposon insertional mutagenesis system from Dr Xu's lab.^24^ The Act‐RFP cassette of the piggyBac transposon element was successfully inserted into the first intron of the *Clmp* gene, resulting in its transcriptional termination (Figure 2A). The successful insertion of the RFP cassette in the first intron of the *Clmp* gene could be confirmed by PCR analysis and RFP fluorescence under a handheld UV lamp (Figure 2B). The qRT‐PCR analysis indicated a 49% mRNA reduction in *Clmp* in the heterozygous mutant hearts (*Clmp* ^+/−^), and complete knockout in the homozygous mutant mice (*Clmp* ^−/−^), which indicates the *Clmp* deficiency is efficient (Figure 2C). Furthermore, we noted that the homozygous mutant (*Clmp* ^−/−^) offspring could not survive more than 4 weeks, which indicates *Clmp* is essential for post‐natal development. We analysed the ratio of three genotypes on day 1 after birth from heterozygous mating. On post‐natal day 1, the percentages of the wild‐type (*Clmp* ^+/+^), *Clmp* ^+/−^ and *Clmp* ^−/−^ mice were 28.9%, 44.4% and 26.7%, respectively, which were consistent with Mendelian inheritance. However, the *Clmp* ^−/−^ mice started to die on post‐natal day 3 and the percentage of survival *Clmp* ^−/−^ mice was decreased to 2.66% at week 3 (Figure 2D), with no *Clmp* ^−/−^ mice surviving more than 4 weeks. The obviously decreased heart size and HW/BW ratios in the homozygous mutant offspring suggest a growth retardation of these mice during post‐natal development (Figure S2A,B). Collectively, these results indicate that *Clmp* knockout significantly decreases the survival rate of mice.

### 3.3 Decreased *Clmp* expression leads to the increased heart injury after MI

**FIGURE 3:**
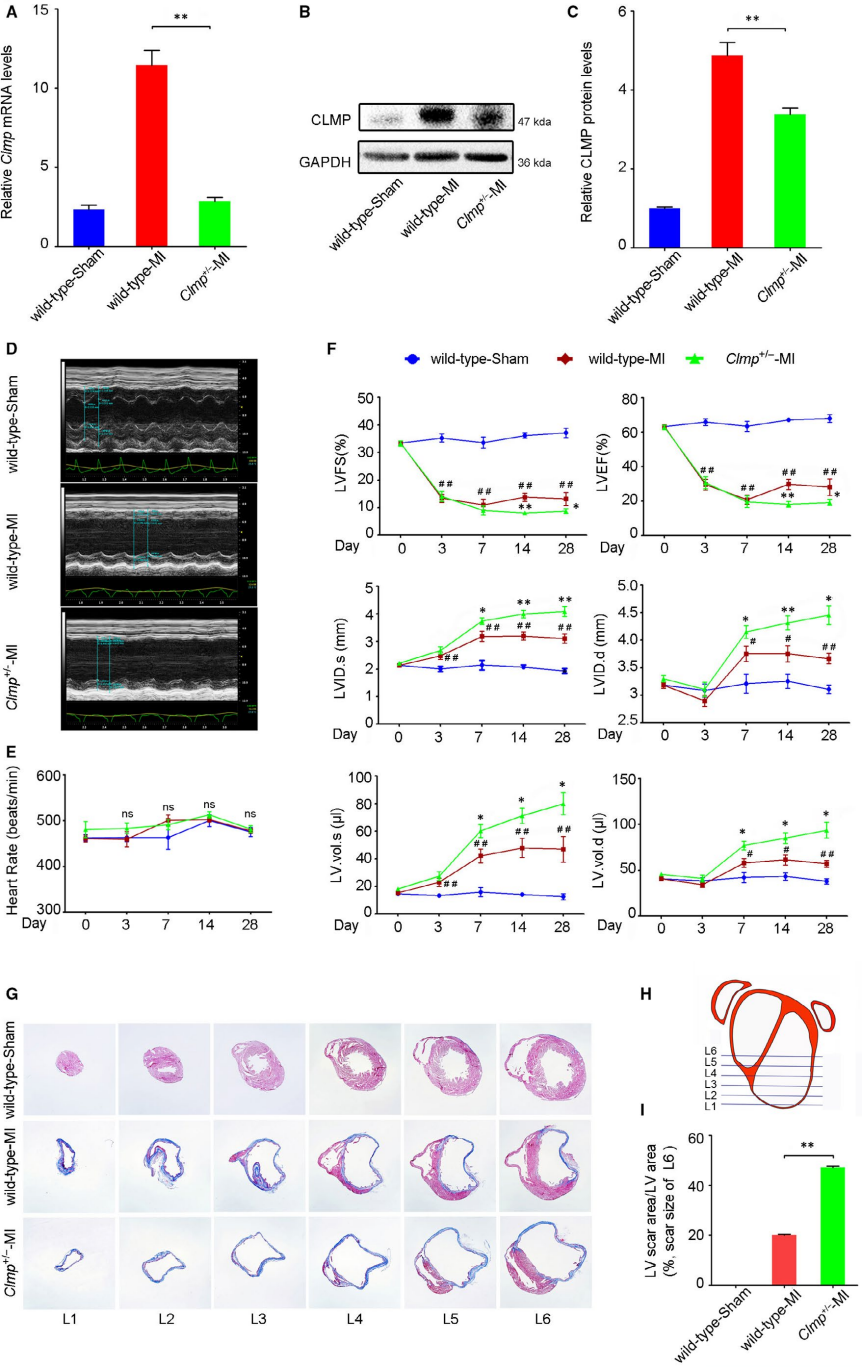
*Clmp* deficiency further deteriorates the ventricular function after MI injury. A, B, Compared with the wild‐type MI LV, *Clmp* ^+/−^ MI LV showed decreased *Clmp* mRNA and protein levels detected by qRT‐PCR and Western blot, respectively. C, The relative densitometric quantification of panel B. D, The representative M‐mode echocardiographic images at week 4 after MI (n = 15 per group) indicated more severe cardiac dysfunction in the *Clmp* ^+/−^ MI mice. E, The heart rates among three groups on days 0, 3, 7, 14 and 28 post‐MI. F, The transthoracic echocardiographic analyses were performed on days 0, 3, 7, 14 and 28 post‐MI. LVFS: left ventricular fractional shortening; LVEF: left ventricular ejection fraction; LVID.s: left ventricular internal dimension at end systole; LVID.d: left ventricular internal dimension at end diastole; LV.vol.s: left ventricular volume at end systole; LV.vol.d: left ventricular volume at end diastole. Two‐way repeated‐measures ANOVA; *P < .05 and **P < .01 (wild‐type MI vs*Clmp* ^+/−^‐MI); ^#^ *P* < .05 and ^##^ *P* < .01 (wild‐type MI vs wild type). G, The representative images of Masson's trichrome in mouse hearts. H, The schematic diagram of serial transverse heart sections for Masson's trichrome staining. I, The quantification of L6 showed a significant increase in fibrosis areas in the *Clmp* ^+/−^ MI heart. All data are presented as the mean ± SEM. One‐way ANOVA; *P < .05; **P < .01

Due to the post‐natal lethality of *Clmp* ^−/−^ mice, *Clmp* ^+/−^ mice were used to further investigate the roles of *Clmp* in the heart. We initially evaluated the heart function of *Clmp* ^+/−^ and wild‐type mice. Comparable values for the heart‐to‐bodyweight ratio, LVEF, LVFS, LV mass and heart rate were observed under the normal physical condition (Figure S2A,C‐G). Furthermore, a series of markers indicating cardiac injury (ANP and BNP), cell proliferation, apoptosis, autophagy, necrosis and pyroptosis were unchanged between the two normal groups (Figure S2H‐P). Meanwhile, oestrogen pre‐treatment process of primary CFs was operated to exclude gender factors (Figure S3A‐D). We then investigated the roles of *Clmp* in MI by LAD ligation of *Clmp* ^+/−^ and wild‐type mice. The mice were randomized into three groups (n = 15 per group): (a) wild‐type Sham group, (b) wild‐type MI group and (c) *Clmp* ^+/−^‐MI group. Using qRT‐PCR and Western blot analyses, we found that the mRNAs and protein levels of CLMP were significantly decreased in the infarcted *Clmp* ^+/−^ mouse hearts (Figure 3A‐C), which indicates the *Clmp* was efficiently knocked down. Cardiac function was examined using two‐dimensional (2D) echocardiography at different time points within 4 weeks (Figure 3D). The heart rates showed no difference among the three groups (Figure 3E). According to the *Clmp* expression pattern in the ischaemic heart, we initially speculated *Clmp* knockdown may improve the cardiac function of MI mice. However, when compared to that in the wild‐type MI animals, we observed significantly lower LVEF and LVFS values in the *Clmp* ^+/−^ MI mice at 2 and 4 weeks post‐MI surgery, indicating worsen ventricular dysfunction of these mice (Figure 3F). Remarkably, the values of left ventricular internal diameter at end diastole and end systole (LVID.s and LVID.d) were significantly increased in the *Clmp* ^+/−^ MI mice, in addition to the LV end‐systolic volume (LV.vol.s) and end‐diastolic volume (LV.vol.d). These results represented a deteriorative heart function accompanied by heart dilation in the *Clmp* ^+/−^ MI mice. At week 4 post‐MI, hearts were collected, and Masson's trichrome staining was performed to analyse cardiac fibrosis (Figure 3G‐I). The area of myocardium fibrosis was calculated as a percentage of the total area of the LV myocardium (Figure S4). As shown in Figure 3G,I, the area of fibrosis in the *Clmp* ^+/−^ MI mice was significantly larger than that in the wild‐type MI mice. Taken together, the results suggest that *Clmp* ^+/−^ mice exhibit more serious cardiac dysfunction after the ligation of LAD.

### 3.4 Knockdown of *Clmp* results in accumulating excessive IL‐1β in the infarcted myocardium

**FIGURE 4:**
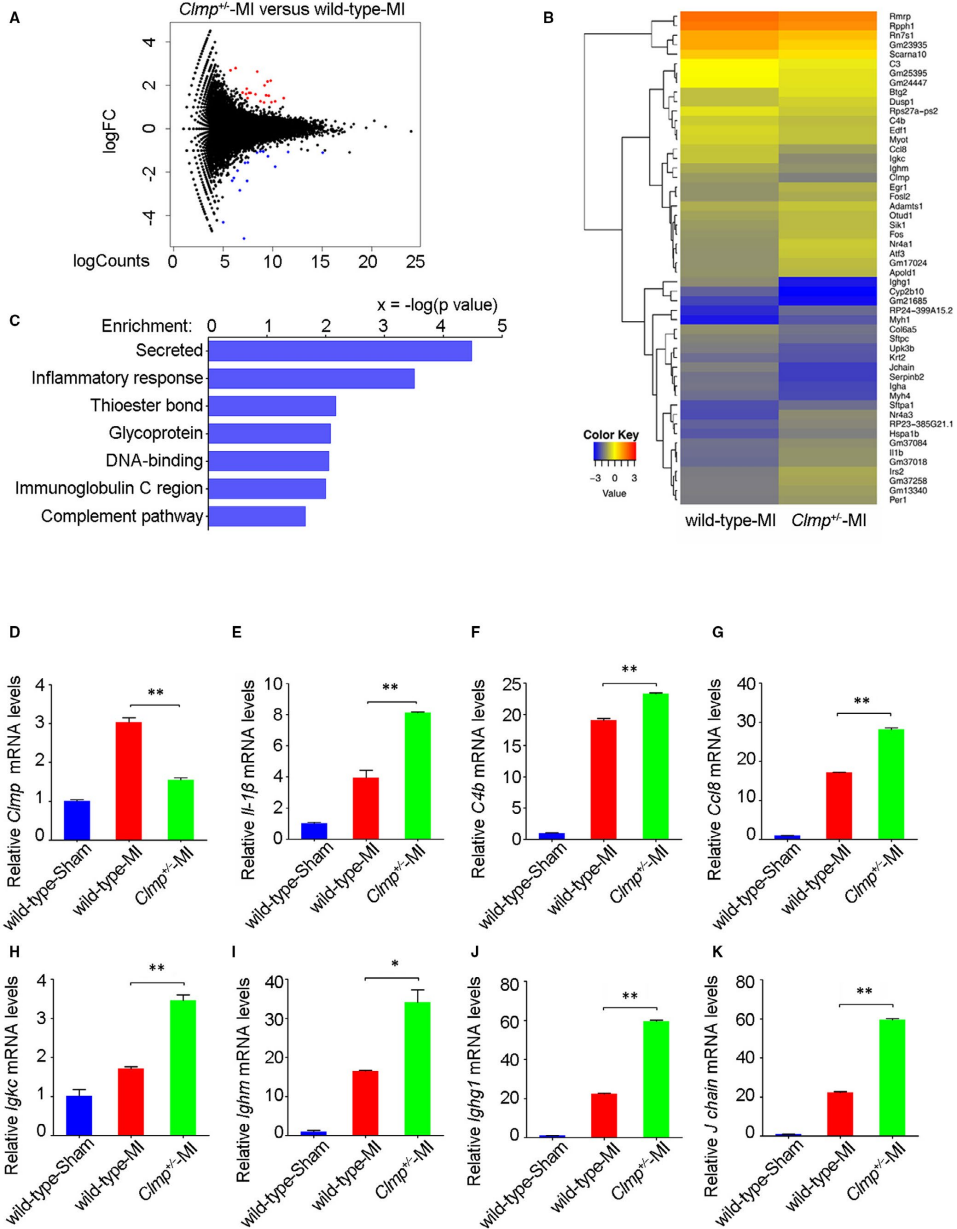
The *Clmp* ^+/−^ mice show excessive accumulation of the inflammatory factors in the infarcted myocardium. A, Through RNA‐Seq analysis, gene expression changes in the MI LV were showed with Volcano Plot by pair comparison (*Clmp* ^+/−^ vs wild‐type, n = 3). Red and blue dots indicate significantly differentially expressed genes. B, The heatmap depicted 52 differentially expressed genes at week 2 after MI between the *Clmp* ^+/−^ and wild‐type mouse hearts. C, The GO term enrichment analysis indicated that the changed genes were mainly involved in the secreted and inflammatory response. D‐K, The qRT‐PCR assays showed the expression levels of *Clmp* and the inflammatory‐associated genes *IL‐1β*, *C4b*, *Ccl8*, *Igkc*, *Ighm*, *Ighg1* and *J chain* in the wild‐type sham, wild‐type MI and *Clmp* ^+/−^ MI LVs. Data are presented as the mean ± SEM. Student's t test or one‐way ANOVA; *P < .05; **P < .01

To investigate the potential mechanism of *Clmp* knockdown‐induced functional deterioration, we performed RNA sequencing (RNA‐Seq) analysis on wild‐type and *Clmp* ^+/−^ LV of the infarcted heart. As shown in Figure 4A,B, we identified 52 differentially expressed genes between the *Clmp* ^+/−^ and wild‐type MI hearts with a fold change >1.5 and *P*‐value <.05. Among these genes, 26 genes were up‐regulated in the *Clmp* ^+/−^ MI LV compared with the wild‐type MI LV (Figure 4B). Functional categories indicated that the changed genes were mainly secreted proteins and functioned in the inflammatory response (Figure 4C), in which IL‐1β was involved in both functional categories. We further evaluated the expression of *Clmp* and inflammatory genes using qRT‐PCR and found that the expression levels of *Il‐1β*, *C4b*, *Ighg1, Ighm, Igkc, J chain and Ccl8* were significantly increased in the *Clmp* ^+/−^ MI heart compared with the wild‐type MI heart (Figure 4D‐K), which indicates an increased inflammatory response triggered in *Clmp* ^+/−^ MI heart. Recent studies have showed pyroptosis was a major style of cell death during the inflammatory period, and pyroptosis has also been shown to aggravate the inflammatory response through triggering IL‐1β production.^5^, ^32^ Therefore, we speculate that pyroptosis may be a main culprit of the increased inflammatory response and cardiac injury in the *Clmp* ^+/−^ MI heart.

### 3.5 Pyroptosis were substantially increased in the infarct zone of *Clmp* ^+/−^ mice

**FIGURE 5:**
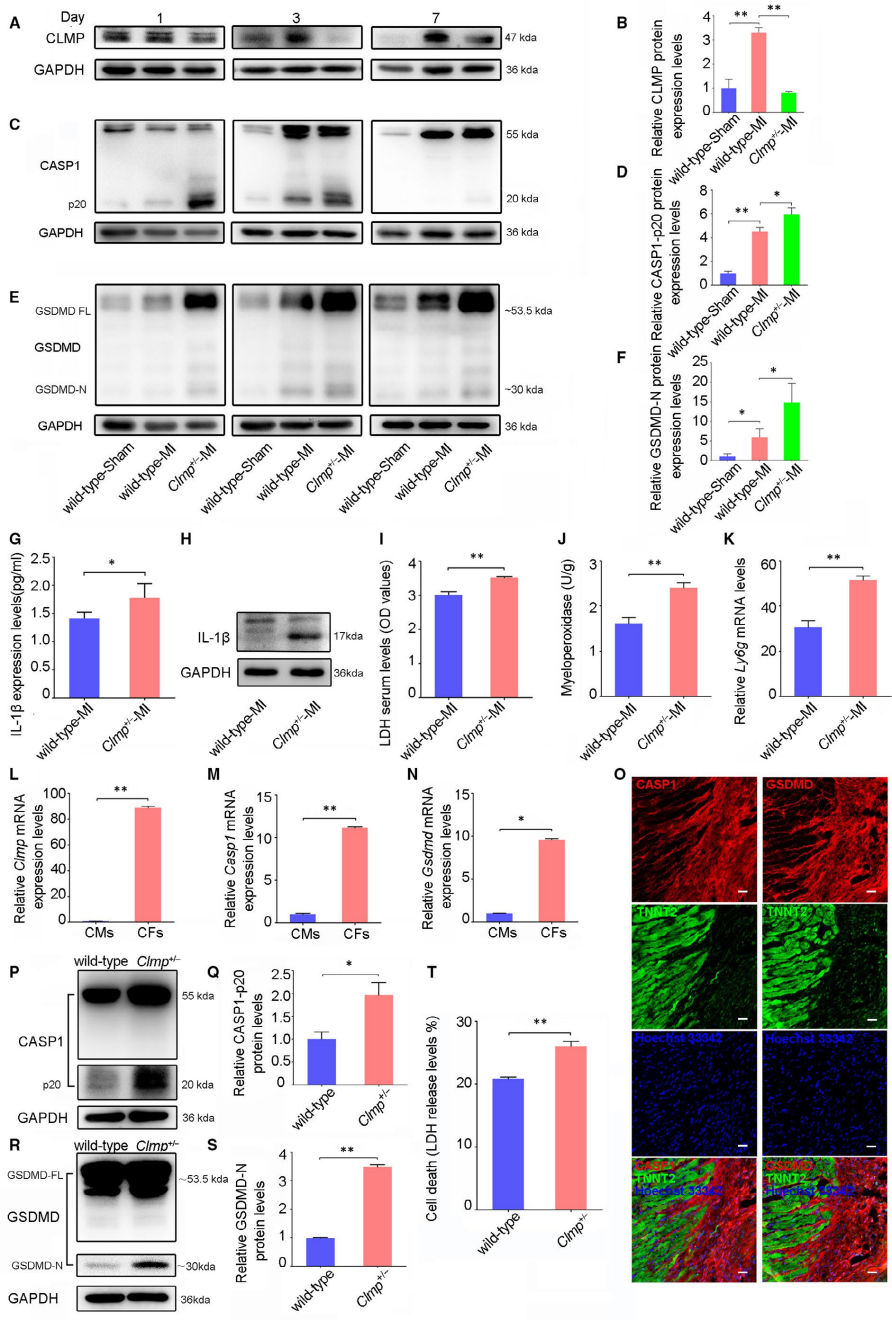
*Clmp* knockdown results in fibroblast pyroptosis and inflammation. A, The representative Western blot imaging of CLMP in the wild‐type sham, wild‐type MI and *Clmp* ^+/−^ MI LVs on the indicated days post‐MI. B, The relative densitometric quantification of CLMP protein on day 3 post‐MI in panel E (n = 3). C, The representative Western blot imaging of CASP1 in the wild‐type sham, wild‐type MI and *Clmp* ^+/−^ MI hearts on the indicated days post‐MI. D,The relative densitometric quantification of active CASP1 (P20) protein on day 3 post‐MI in panel A (n = 3). E,The representative Western blot imaging of GSDMD in the wild‐type sham, wild‐type MI and *Clmp* ^+/−^ MI LVs on the indicated days post‐MI. GSDMD‐FL, GSDMD full‐length; GSDMD‐N, GSDMD cleavage. F, The relative densitometric quantification of GSDMD‐N protein on day 3 post‐MI in panel C (n = 3). G, IL‐1β expression in the wild‐type MI and *Clmp* ^+/−^ MI mice from ELISA. H, Cleaved IL‐1β protein by Western blot analysis. I, The serum LDH concentrations in the wild‐type MI and *Clmp* ^+/−^ MI mice on day 3 post‐MI (wild‐type: n = 5; *Clmp* ^+/−^: n = 4). J, The myeloperoxidase analysis in wild‐type MI and *Clmp* ^+/−^ MI LV (wild‐type: n = 5; *Clmp* ^+/−^: n = 4). K, The qRT‐PCR analysis of *Ly6g* among the wild‐type sham, wild‐type MI and *Clmp* ^+/−^ MI LV (wild‐type: n = 3; *Clmp* ^+/−^: n = 3. L‐N, The qRT‐PCR analysis of *Clmp*, *Casp1* and *Gsdmd* in the adult cardiac fibroblasts (CFs) and cardiomyocytes (CMs). O, The representative images of immunostaining for CASP1, GSDMD and TNNT2 in the ischaemic hearts. Hoechst 33342 was used to label nuclei. Scale bar indicates 100 μm. P, The representative Western blot imaging of CASP1 in isolated adult wild‐type and *Clmp* ^+/−^ CFs with pyroptosis induction. Q, The relative densitometric quantification of panel L. R, The representative Western blot imaging of GSDMD in the isolated adult wild‐type and *Clmp* ^+/−^ CFs with pyroptosis induction. S, The relative densitometric quantification of panel N. T, The levels of LDH in the supernatant of isolated adult wild‐type and *Clmp* ^+/−^ fibroblasts after pyroptosis induction (wild‐type: n = 5; *Clmp* ^+/−^: n = 4). All data are presented as the mean ± SEM. Student's *t* test or one‐way ANOVA; *P < .05; **P < .01

In general, inflammation response was believed to occur in early stage of MI. However, excessive accumulation of IL‐1β at 2 weeks post‐MI indicated that excessive inflammatory response may appear in early stage of MI. Therefore, we further researched the earlier period of MI. The Western blot analysis on CLMP expression showed a rising trend at different time points, which was consistent with above‐mentioned results after MI (Figure 5A,B). To investigate whether *Clmp* knockdown could induce pyroptosis in the *Clmp* ^+/−^ MI heart, we analysed the protein expression of pyroptotic mediators, caspase‐1 (CASP1) and GSDMD, during the inflammatory response phase of rodent MI (day 1, 3 and 7 post‐MI). The *Clmp* ^+/−^ LV showed an increased protein level of cleaved CASP1 (p20), compared with the wild‐type LV on day 1 and 3 post‐MI (Figure 5C,D). The cleaved GSDMD (GSDMD‐N) protein showed the similar trends as the cleaved CASP1 (Figure 5E,F), which confirms the increased pyroptosis in *Clmp* ^+/−^ MI LV. We also assessed the level of cell necrosis, another inflammatory forms of PCD, in the infarcted heart tissues. However, no obvious change in necrosis markers (RIP3 and CaMKII) was observed in the *Clmp* ^+/−^ MI LV compared with the wild‐type MI LV (Figure S5A‐D). In addition, the expression levels of cell apoptosis marker PARP were comparable in both WT‐MI and *Clmp* ^+/−^‐MI hearts on day 3 post‐MI (Figure S5E,F). As an integral part of inflammasome, NLRP3 has an important role in sensing “danger factor.” However, the protein levels of NLRP3 were comparable between WT‐MI and *Clmp* ^+/−^‐MI mice (Figure S5G‐I). These data indicate CLMP mainly participates in pyroptosis at the inflammatory stage post‐ MI.

As an important factor, IL‐1β protein expression in myocardium was detected by ELISA and Western blot. We further confirmed that cleaved IL‐β protein expression and were increased obviously in *Clmp* ^+/−^‐MI group at the early stage after acute MI (Figure 5G,H). The release of LDH has been regarded as an indicator of cell death, which was also used to evaluate the degree of pyroptosis.^30^, ^33^ We measured the concentrations of LDH in the sera and found that the *Clmp* ^+/−^ MI mice released significantly higher levels of LDH than the wild‐type MI mice (Figure 5I), which was consistent with the deteriorated cardiac injury in the *Clmp* ^+/−^ MI mice. Inflammation was always accompanied by the recruitment of leucocytes (particularly neutrophils). Our data subsequently showed that the myeloperoxidase (MPO)‐quantified accumulation of neutrophils was significantly increased in the ischaemic cardiac tissues of the *Clmp* ^+/−^ mice (Figure 5J). The expression of *Ly6g*, a marker of neutrophils, was also elevated in the *Clmp* ^+/−^ MI hearts (Figure 5K). Taken together, the knockdown of *Clmp* leads to more serious myocardial injury through promoting pyroptosis and subsequent neutrophil accumulation. However, further studies were needed to determine the cell types undergoing pyroptosis post‐MI.

### 3.6 *Clmp* knockdown promotes inflammatory response via cardiac fibroblast pyroptosis

Considering the specific expression of *Clmp* in fibroblasts, we assumed pyroptosis might occur in CFs. The qRT‐PCR analysis indicated that *Casp1* and *Gsdmd* mRNAs were highly enriched in adult CFs (Figure 5L‐N). Furthermore, we observed that these proteins were localized in the CFs rather than in the TNNT2‐positive cardiomyocytes in vivo (Figure 5O). Thus, CLMP might be responsible for the inhibition of pyroptosis in CFs and the subsequent prevention of excessive inflammation.

**FIGURE 6:**
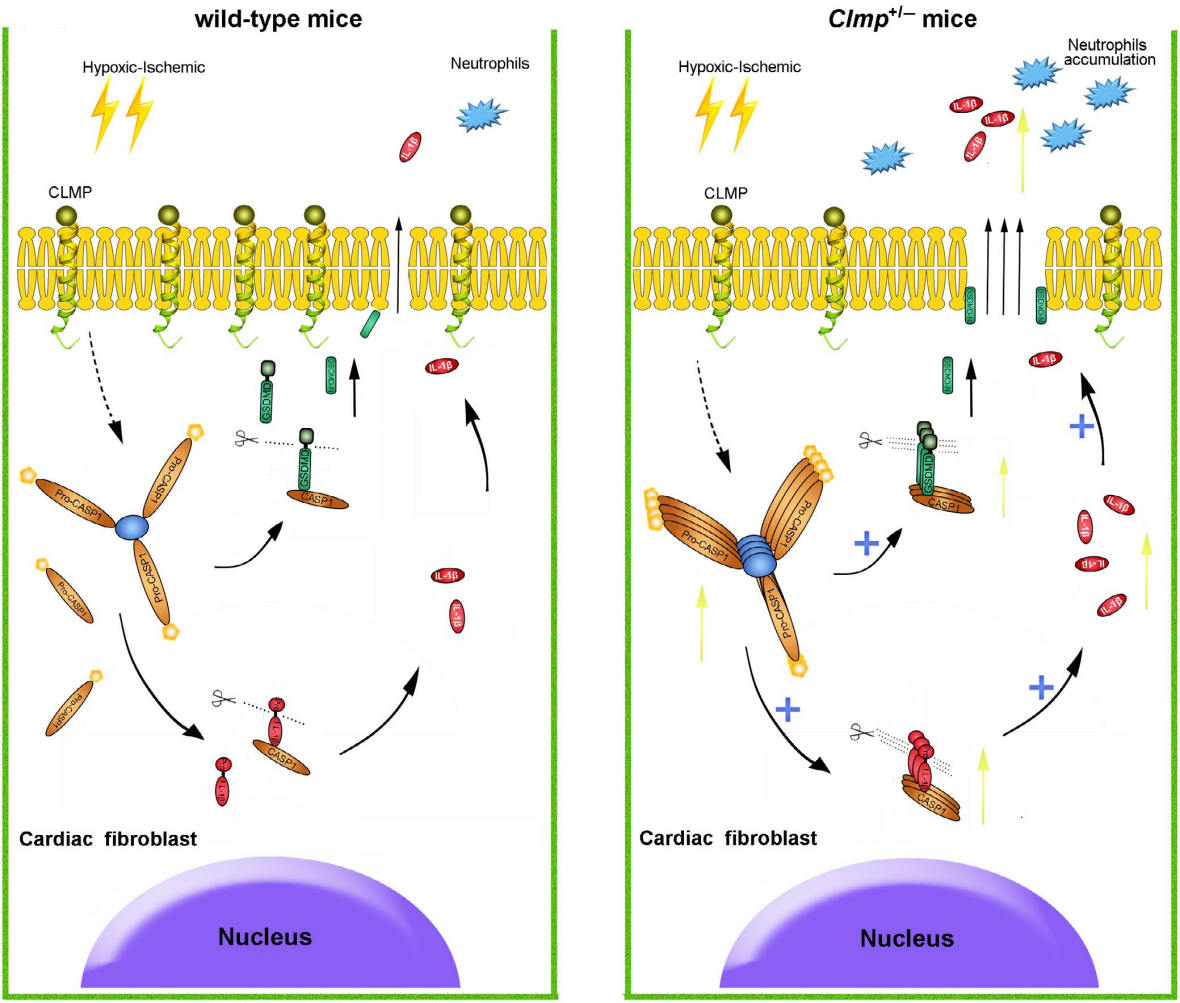
Working model of pyroptosis caused by *Clmp* knockdown in cardiac fibroblasts after MI *Clmp* deficiency may induce the self‐assembly of inflammasome triggered by myocardial ischaemia. The activation of inflammasome promoted the maturation of CASP1 protein from pro‐CASP1. On the one hand, matured CASP1 induced the cleavage of pro‐interleukin 1β (pro‐IL‐1β) to IL‐1β. On the other hand, GSDMD was cleaved by CASP1 in the inflammasome and promoted the release of IL‐1β by forming pores in the plasma membrane. Excessive IL‐1β released into the surrounding environment in short period of time and aggravated the recruitment of neutrophils. Excessive IL‐1β could also trigger severe inflammation response and led to a more severe myocardial injury

As previously described, LPS plus nigericin was used to activate inflammasome with features of caspase‐1‐mediated pyroptosis and IL‐1β secretion.^33^, ^34^ We subsequently compared the sensitivity of CFs and cardiomyocytes to LPS plus nigericin‐induced‐pyroptosis. *Clmp*, *Il‐1β* and *Tnf‐α* showed no significant change in cardiomyocytes after LPS plus nigericin treatment (Figure S6A), while *Casp1* and *Il‐6* were undetectable both before pyroptosis induction and after pyroptosis induction. In contrast, LPS plus nigericin treatment significantly induced the expression levels of *Clmp*, the pyroptosis‐related gene *Casp1*, and the inflammation‐related genes *Il‐1β*, *Il‐6* and *Tnf‐α* in CFs (Figure S6B). Moreover, CFs isolated from the *Clmp* ^+/−^ mice showed substantially more pyroptosis after LPS plus nigericin treatment, as indicated by increased expression of cleaved CASP1 (Figure 5P,Q) and GSDMD proteins (Figure 5R,S) in the *Clmp* ^+/−^ fibroblasts. The LDH level was also significantly higher in the cell culture supernatant of the *Clmp* ^+/−^ fibroblasts than that in the wild‐type fibroblasts (25.97% vs 20.80%) (Figure 5T). Collectively, our results show that more serious pyroptosis occurring in *Clmp* ^+/−^ CFs leads to excessive IL‐1β production, which aggravates the inflammatory response and results in cell death and tissue damage (Figure 6).

## 4 DISCUSSION

As a type I transmembrane protein, CLMP localizes to junctional complexes and may have a role in cell‐cell adhesion. Previous studies have showed it may play important roles in adipocyte maturation, spermatogenesis and intestinal development.^20^, ^22^, ^35^ Loss‐of‐function mutations in CLMP were found in patients with congenital short‐bowel syndrome.^22^ In this study, we found that CLMP expression showed heart injury‐induced increase in fibroblasts from the ischaemic zone. However, the decreased expression of CLMP through gene targeting resulted in more severe heart dysfunction after MI, which indicates its protective role after heart injury. Thus, we demonstrated the new function of CLMP under heart pathology. Meanwhile, our data proved that a decrease in CLMP levels to 50% worsens outcome. Single nucleotide polymorphism in human may result in differential CLMP expression levels and affect the degree of heart injury. Thus, we provide a molecular target for interpretation of MI in human as well.

Ischaemia could trigger a protective immune response and contribute to clearance of damaged cells and ECM. It is necessary to initiate self‐healing mechanisms.^14^ However, a disproportionately excessive inflammatory response can lead to sustained tissue damage and improper healing. Therefore, the balance of inflammation after MI is important for heart protection from injury. Accumulating evidences have suggested that appropriate intervention of the inflammatory response was beneficial in the treatment of MI.^36^ Many mediators have been implicated in the suppression of the post‐infarction inflammatory reaction.^37^, ^38^ In the early inflammatory response stage, CFs are characterized by a pro‐inflammatory phenotype and are capable of secreting a large number of pro‐inflammatory mediators, including IL‐1β.^39^ Animal experiments have shown that enhanced IL‐1β expression had deleterious effects on cardiac function.^40^ Recent research proved that Canakinumab, as an IL‐1β inhibitor, provided a novel approach to treat heart failure post‐MI.^41^ In this study, *Clmp* knockdown mice showed increased accumulation of IL‐1β and infiltration of inflammatory cells in the infarct area, which indicates excessive inflammation, are the reasons for increased heart injury. Thus, CLMP was important for the balance of the inflammatory response after MI.

Pyroptosis and necrosis are both inflammatory PCD. We found pyroptosis, but not necrosis, was significantly increased in *Clmp* ^+/−^ MI hearts compared with wild‐type MI hearts, as evidenced by substantial increases in cleaved GSDMD and CASP1. Thus, pyroptosis‐mediated inflammasome may play a crucial role in generating IL‐1β and initiating inflammatory responses in the MI heart.^18^, ^42^ Furthermore, we noted that CLMP, GSDMD and CASP1 were specifically increased in CFs after ischaemic myocardial damage, which indicates pyroptosis may mainly occur in fibroblasts. We further confirmed the increased cleavage of GSDMD and CASP1 in isolated fibroblasts after pyroptosis induction. These results were supported by a previous study that demonstrated inflammasome was activated in CFs, but not in cardiomyocytes after myocardial I/R injury.^18^ In this study, we identified the fibroblast‐expressed CLMP as a brake of pyroptosis to balance the heart inflammatory response and protect heart function.

Inflammation was thought to be responsible for the resorption of the wound and tissue regeneration. Inflammasome is a key component sensing danger factor and triggering a local or systemic inflammatory reaction. The inflammasome is a macromolecular protein complex, which contains NOD‐like receptors (NLRs), inflammatory caspases (pro‐caspase‐1) and apoptosis‐associated speck‐like protein containing a CARD (ASC).^43^, ^44^ Activation of the inflammasome is a key function mediated by the innate immune system and has been linked to various cardiovascular diseases, including endothelial injury, atherosclerosis, MI and hypertension.^45^, ^46^, ^47^, ^48^ When facing danger‐signalling stimuli, the inflammasome is assembled and ASC molecules aggregate. ASC bridged pro‐caspase‐1 and pyrin‐containing receptors, such as NLRP3. Furthermore, ASC could further lead to the activation of pro‐caspase‐1. In the canonical inflammasome pathway, activated caspase‐1 could process the precursors of IL‐1β and GSDMD to their matured forms, thus ultimately leading to pyroptosis. Pyroptosis may play an important role in the progression of cardiovascular diseases and could be a potential therapeutic intervention target. Increased cleavage of GSDMD and CASP1 in *Clmp* ^+/−^ MI LV showed CLMP functions as an upstream gene of these molecules. However, it remains challenging to identify the precise mechanism of CLMP during pyroptosis and is worth further investigation. In addition, although NLRP3 was not changed at protein level between WT‐MI and *Clmp* ^+/−^ heart in our study, its role in inflammasome activation should be further investigated in the future. As a transmembrane protein, CLMP is composed of an extracellular part (373 amino acids, a V‐ and a C2‐type domain), a transmembrane region (22 amino acids) and a cytoplasmic tail (118 amino acids). The extracellular part may be involved in cell‐cell adhesion; however, the function of the large cytoplasmic tail remains unknown. Deciphering the function of the cytoplasmic region may help identify the mechanism of CLMP in pyroptosis.

In conclusion, our data demonstrated that an insufficient increase in CLMP expression will lead to more serious cardiac dysfunction in the infarcted hearts, partially due to the unbridled fibroblast pyroptosis and excessive inflammation. These results strongly indicated ischaemia‐induced CLMP expression in fibroblasts is crucial for the balance of the inflammatory reaction, providing a novel pyroptotic insight into ischaemic heart diseases and holding substantial potential for the treatment of MI.

## CONFLICT OF INTEREST

The authors declare that they have no conflict of interest.

## AUTHOR CONTRIBUTIONS

**Xinglong Han:** Conceptualization (equal); Formal analysis (equal); Investigation (equal); Methodology (equal); Writing‐original draft (equal); Writing‐review & editing (equal). **Zhen‐Ao Zhao:** Conceptualization (equal); Formal analysis (equal); Investigation (equal); Methodology (equal); Validation (equal); Visualization (equal); Writing‐review & editing (equal). **Shiping Yan:** Data curation (equal); Formal analysis (equal); Investigation (equal); Methodology (equal); Validation (equal). **Wei Lei:** Conceptualization (equal); Data curation (equal); Formal analysis (equal); Funding acquisition (equal); Investigation (equal); Validation (equal); Writing‐review & editing (equal). **Hongchun Wu:** Data curation (equal); Investigation (equal); Methodology (equal). **Xing‐Ai Lu:** Data curation (equal); Investigation (equal); Methodology (equal). **Yueqiu Chen:** Investigation (equal); Methodology (equal); Visualization (equal). **Jingjing Li:** Data curation (equal); Formal analysis (equal); Investigation (equal); Methodology (equal). **Yaning Wang:** Data curation (equal); Investigation (equal); Methodology (equal). **Miao Yu:** Data curation (equal); Investigation (equal); Methodology (equal); Writing‐review & editing (equal). **Yongming Wang:** Conceptualization (equal); Methodology (equal); Project administration (equal); Resources (equal). **Yufang Zheng:** Conceptualization (equal); Data curation (equal); Formal analysis (equal); Investigation (equal); Resources (equal). **Hongyan Wang:** Conceptualization (equal); Funding acquisition (equal); Project administration (equal); Resources (equal); Writing‐review & editing (equal). **Zhenya Shen:** Conceptualization (equal); Funding acquisition (equal); Investigation (equal); Writing‐review & editing (equal). **Shijun Hu:** Conceptualization (equal); Funding acquisition (equal); Investigation (equal); Project administration (equal); Supervision (equal); Visualization (equal); Writing‐review & editing (equal).

## Supporting information

Supplementary Material

## Data Availability

The data that support the findings of this study are available from the corresponding author upon reasonable request.
