# Supplementary material for: CXADR‐like membrane protein protects against heart injury by preventing excessive pyroptosis after myocardial infarction
Source: J Cell Mol Med. 2020 Oct 21;24(23):13775–88. doi: 10.1111/jcmm.15955 (PMC7753842; doi:10.1111/jcmm.15955)
Supplement: Supplementary file 1 — Supplementary Material [file JCMM-24-13775-s001.pdf]

SUPPLEMENTAL IMFORMATION

CXADR-like membrane protein protects against heart injury by preventing excessive pyroptosis after myocardial infarction

Xinglong Han[1, #], Zhen-Ao Zhao[2, 3, #], Shiping Yan[1, #], Wei Lei[1, #], Hongchun Wu[1], Xing-Ai Lu[1], Yueqiu Chen[1], Jingjing Li[1], Miao Yu[1], Yongming Wang[4], Yufang Zheng[5, 6], Hongyan Wang[5, 7, 8], Zhenya Shen[1 *], Shijun Hu[1, *]

[1]Department of Cardiovascular Surgery of the First Affiliated Hospital & Institute for Cardiovascular Science, State Key Laboratory of Radiation Medicine and Protection, Medical College, Soochow University, Suzhou 215000, China; [2]Institute of Microcirculation & Department of Pathophysiology of Basic Medical College, Hebei North University, Zhangjiakou 075000, Hebei, China; [3]Hebei Key Laboratory of Critical Disease Mechanism and Intervention, Zhangjiakou 075000, Hebei, China; [4]MOE Key Laboratory of Contemporary Anthropology at School of Life Sciences and Zhongshan Hospital, Fudan University, Shanghai 200438, China; [5]Obstetrics and Gynecology Hospital, State Key Laboratory of Genetic Engineering at School of Life Sciences, Institute of Reproduction and Development, Fudan University, Shanghai 200011, China; [6]Institute of Developmental Biology & Molecular Medicine, Fudan University, Shanghai 200433, China; [7]Key Laboratory of Reproduction Regulation of NPFPC, Collaborative Innovation Center of Genetics and Development, Fudan University, Shanghai 200032, China; [8]Children's Hospital of Fudan University, Shanghai 201102, China.

[#] Co-first authors

SUPPLEMENTAL MATERIALS AND METHODS

Cell culture. Primary cardiac fibroblasts were isolated from adult mice. Briefly, after dissection, the hearts were detached, washed and minced in a sterile centrifuge tube. Tissues were then digested in basic medium containing 0.1% collagenase II (Sigma, Cat. No. V900892, USA) for 3-5 times until fully digested. The first digestion was discarded. Subsequent supernatants were collected and centrifuged at 200 × g for 5 minutes. Cells were resuspended in DMEM (Gibco, Cat. No. C11995500CP, USA) supplemented with 10% fetal bovine serum (Bio-Ind, Cat. No. 04-001-1A, ISR), 100 U/ mL penicillin and 100 mg/ mL streptomycin. The cell suspension was seeded in a culture dish for fibroblast attachment. After a 2-hour culture, the non-adherent myocytes were removed by medium changing [1]. Adult mouse cardiomyocytes (CMs) were isolated via the Langendorff perfusion/collagenase-digestion procedure as previously described [2]. Briefly, adult mouse hearts were detached, and the coronary arteries were perfused via the aorta at 4 mL/min (37°C) for 5 minutes with a modified Isolation Krebs-Henseleit (MIKH) solution with pH 7.4. The detached hearts were perfused with 0.1% type II collagenase dissolved in MIKH solution [3]. After 15 minutes perfusion, the left ventricular free wall was then cut into strips and mixed to yield a single-cell suspension in MIKH solution containing 0.7% BSA (Sigma, Cat. No. V900933, USA). The Ca^{2+} concentration in the solution was increased in this suspension via stepwise increments until 1 mmol/L. The cells were then resuspended in complete medium (DMEM, 10% FBS, 100 U/ mL penicillin, 100 mg/ mL streptomycin, 1 mmol/L Ca^{2+}, and 10 mM Norepinephrine (Sigma, Cat. No. A0937-1G, USA)), and seeded in a sterile dish. In addition, NIH3T3, H9C2 and C2C12 cells were cultured in DMEM with 10% FBS for in vitro studies and were passaged every 4-6 days using 0.25% Trypsin-EDTA. As previously described [4], HL-1 cell line was cultured in the Claycomb medium (Sigma, Cat. No. 51800C, USA) supplemented with 10% FBS, 10 μM Norepinephrine, 100 units/ml penicillin and 100 mg/ml streptomycin. The C2C12 cells were purchased from Chinese Academy of Sciences Institute of Life Sciences Cell Resource Center (Shanghai, China). The commercial Myco-Blue Mycoplasma Detector (Vazyme, Cat. No. D101, China) was used for mycoplasma test regularly.

Quantitative real-time PCR analysis (qRT-PCR). Total RNAs were extracted using Invitrogen™

TRIzol™ reagent (Thermo Fisher, Cat. No. 15596018, USA). After DNase I digestion, RNAs were reverse transcribed with a TaKaRa PrimeScript™ RT Reagent Kit (Clontech, Cat. No. rr037b, USA). RNA expression was detected and analyzed using the Applied Biosystems StepOnePlus™ Real-Time PCR System (Thermo Fisher, USA). The primers used in this study are listed in **Supplementary Table 1**.

Western blot. Left ventricle (LV) cardiac tissues or cells were lysed in RIPA lysis buffer with protease and phosphatase cocktail inhibitors. The protein concentration was determined using a Pierce™ BCA Protein Assay Kit (Thermo Fisher, Cat. No. 23227, USA), and a total of 15 μg protein was loaded for each sample. The proteins were separated using 10% SDS-PAGE gels at 100 V for ~1.5 hours. Proteins were transferred to a 0.45 μm PVDF membrane (Millipore, IPVH00010) in ice-cold transfer buffer at 200 mA for 1.5-2 hours. After blocked in 5% skimmed milk (dissolved in TBS-0.1% Tween 20), the membrane was incubated with appropriate primary antibodies: anti-CLMP (1:1000), anti-GSDMD (1:1000), anti-RIP3 (1:1000), anti-CaMKII (1:2000), anti-caspase-1 (1:1000)， anti-PARP (1:1000), anti-IL-1β (1:1000), anti-p62 (1:1000), anti-p-histone H3 (1:1000), anti-total histone H3 (1:1000), anti-NLRP3 (1:1000) and anti-GAPDH (1:1000) respectively. After three washes in TBS-T buffer, the membranes were incubated with 1:3000 (HRP)-conjugated secondary antibodies for 1 hour at room temperature. Protein detection was performed using chemiluminescent substrates (Merck Millipore, Cat. No. WBKLS0500, Germany). All the western blot tests were repeated for 3 times. Quantification was performed by ImageJ software. The antibodies used in this study are listed in **Supplementary Table 2.**

Immunofluorescence. Mouse hearts were embedded in OCT compound (Sakura, Cat. No. k4583, USA) and frozen in liquid nitrogen. Tissue sections or cell slides were permeabilized with 0.5% Triton X-100 in PBS for 5 minutes at room temperature followed by incubation with 5% BSA in PBST (PBS containing 0.1% Tween 20) for 30 minutes. Tissue sections or cell slides were incubated with primary antibodies in a humidified atmosphere for 2 hours at room temperature or overnight at 4°C. After washing 3 times for 15 minutes with PBST, the sections were incubated with fluorescent secondary antibodies for 1 hour in a humid chamber, followed by nuclear staining with Hoechst 33342. The sections or slides were covered with cover

slips using fluorescent mounting medium. Images were obtained at 25°C using the LSM 880 Confocal Laser Scanning Microscope (Zeiss, Germany).

Masson's trichrome staining. The hearts from 5 mice of each group were collected randomly at week 4 after MI. Six sections with 7-μm thickness were made at 540-μm intervals and collected for fibrosis staining with Masson's trichrome following the kit (Sigma, Cat. No. ht15-1kt, USA). Briefly, the slides were immersed in preheated Bouin's Solution at 56°C for 15 minutes, rinsed with deionized water to remove yellow color from sections. And then, the slides were placed sequentially in weigert's iron hematoxylin solution, biebrich scarlet-acid fuchsin, phosphotungstic/phosphomolybdic acid solution and aniline blue solution for 5 minutes each time. Finally, the sections were washed by deionized water, dehydrated through graded ethanol, cleared in xylene and mounted. To quantify fibrosis, the stained area was measured and normalized to the total area of the left ventricle at the sixth transversal level using ImageJ software.

RNA sequencing (RNA-Seq). The ischemic myocardium of the left ventricle was obtained at week 2 post-MI. Total RNAs were extracted with Invitrogen^{TM} TRIzol^{TM} reagent (Thermo Fisher, Cat. No. 15596018, USA). RNA-Seq library preparation and deep sequencing were performed by Genewiz (China). Transcript-level expression analysis of RNA-Seq was operated with Hierarchical Indexing for Spliced Alignment of Transcripts (Hisat2, USA) in Novogene (China).

SUPPLEMENTAL FIGURES

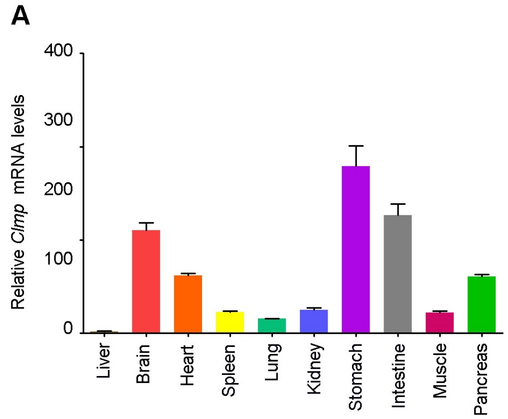

Supplemental Figure 1. Comparison of *Clmp* mRNA levels in different adult tissues in mice.

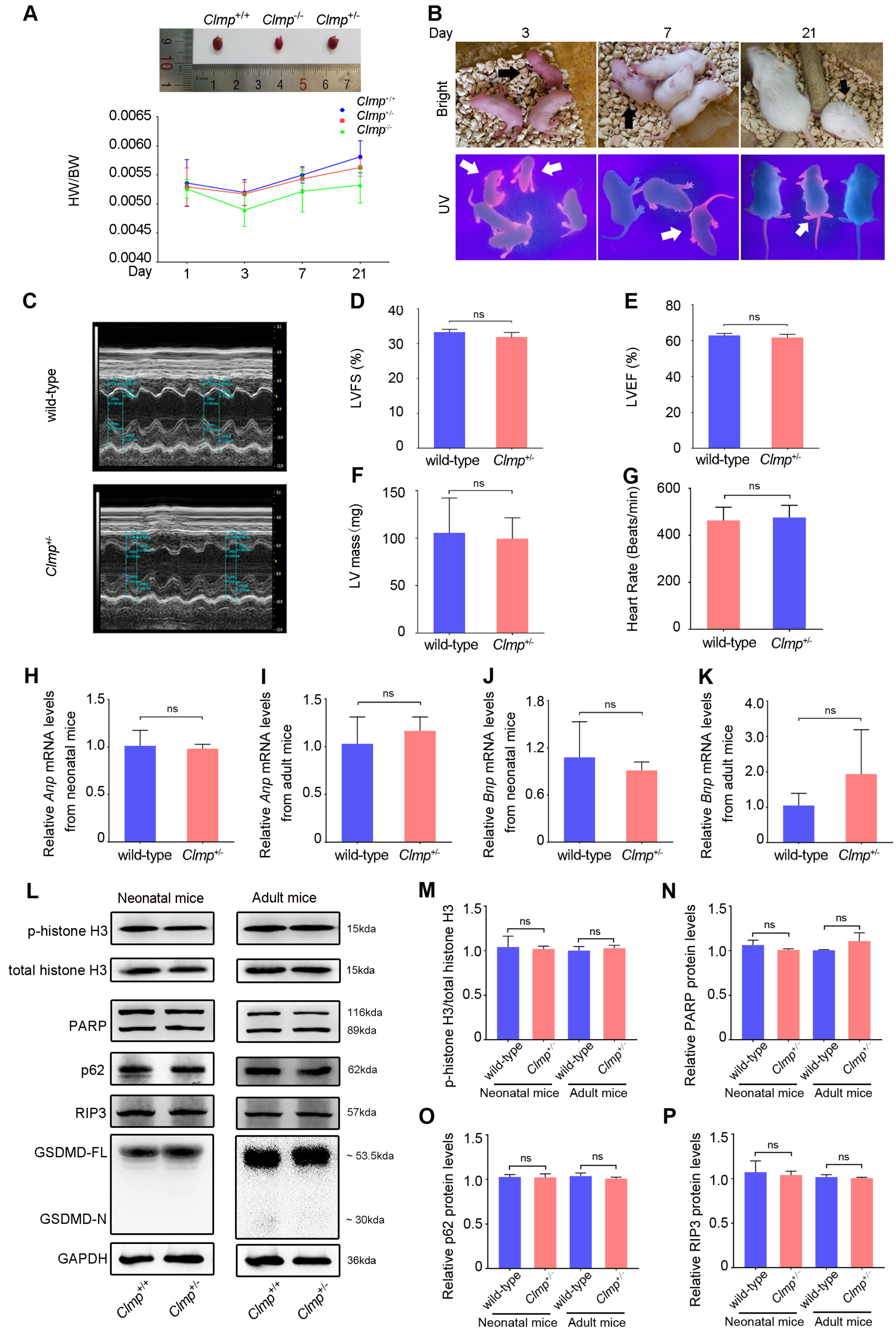

A
B
C
D
E
F
G
H
I
J
K
L
M
N
O
P
Clmp+/+ Clmp-/- Clmp+/-
HW/BW
Day
Bright
UV
wild-type
Clmp+/-
LVFS (%)
LVEF (%)
LV mass (mg)
Heart Rate (Beats/min)
ns
Relative Anp mRNA levels from neonatal mice
Relative Anp mRNA levels from adult mice
Relative Bnp mRNA levels from neonatal mice
Relative Bnp mRNA levels from adult mice
Neonatal mice
Adult mice
p-histone H3
total histone H3
PARP
p62
RIP3
GSDMD-FL
GSDMD-N
GAPDH
15kda
116kda
89kda
62kda
57kda
~ 53.5kda
~ 30kda
36kda
p-histone H3/total histone H3
Relative PARP protein levels
Relative p62 protein levels
Relative RIP3 protein levels

96

Supplemental Figure 2. *Clmp* knockdown does not affect cardiac function of the adult mice. (**A**) Assessment of the heart-to-body weight ratio. HW: heart weight, BW: body weight. (**B**) The homozygous mutant offspring (White arrow and black arrow) showed growth retardation during postnatal development. (**C**) The representative M-mode echocardiographic images of the wild-type and $Clmp^{+/-}$ mice. (**D**) The quantification of the left ventricle fractional shortening (LVFS) between the wild-type and $Clmp^{+/-}$ mice. (**E**) The quantification of the left ventricle ejection fraction (LVEF) between the wild-type and $Clmp^{+/-}$ mice. **(F)** The quantification of the left ventricle mass between the wild-type and $Clmp^{+/-}$ mice. **(G)** The heart rates of the wild-type and $Clmp^{+/-}$ mice. (**H**-**K**) ANP and BNP mRNA levels in neonatal and adult mice between the wild-type and $Clmp^{+/-}$ mice. (**L**) PARP, p62, RIP3, GSDMD and p-histone H3 protein levels between the wild-type and $Clmp^{+/-}$ mice. (**M**-**P**) The quantification of p-histone H3/total histone H3, PARP, p62 and RIP3 in the wild-type and $Clmp^{+/-}$ hearts.

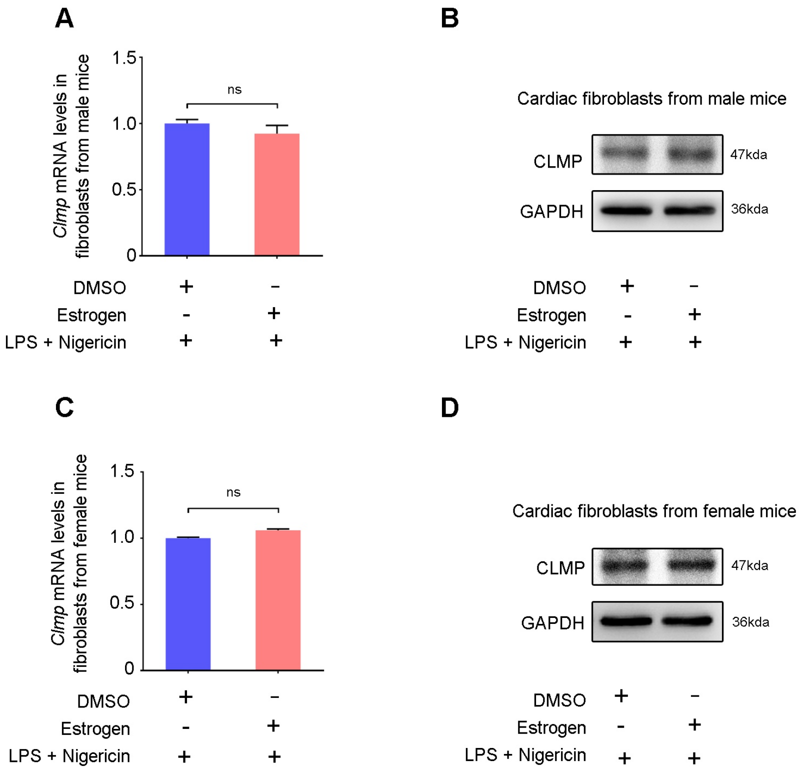

Supplemental Figure 3. **CLMP expression was not affected by treating with estrogen in primary fibroblasts from male and female mice**. (**A** & **B**) The mRNA and protein levels of CLMP from male primary fibroblasts in the estrogen treatment and normal group. (**C** & **D**) The mRNA and protein levels of CLMP from female primary fibroblasts in the estrogen treatment and normal group.

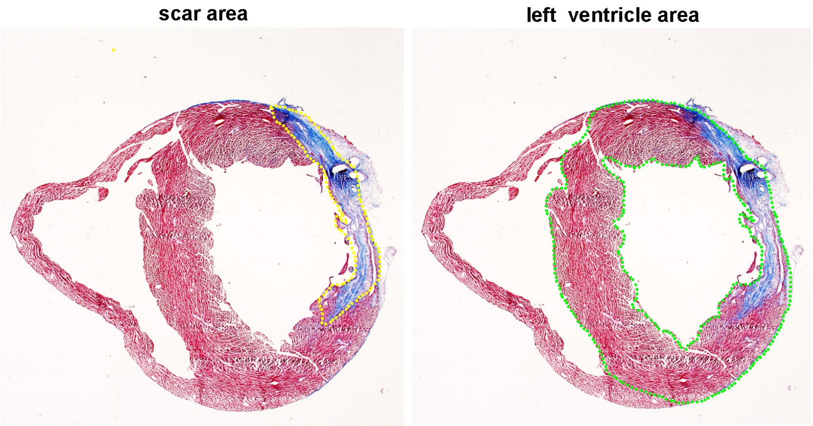

Supplemental Figure 4. Method for quantification of scar area as percentage of the transversal ventricle area.

The quantitative analysis of the left ventricular (LV) scar area by Masson's trichrome staining at week 4 after myocardial infarction. The scar area was labelled with the yellow dotted line in the left panel and the LV area was labelled with the green dotted line in the right panel. The percentage of LV scar area (%) = LV scar area (yellow) / LV area (green).

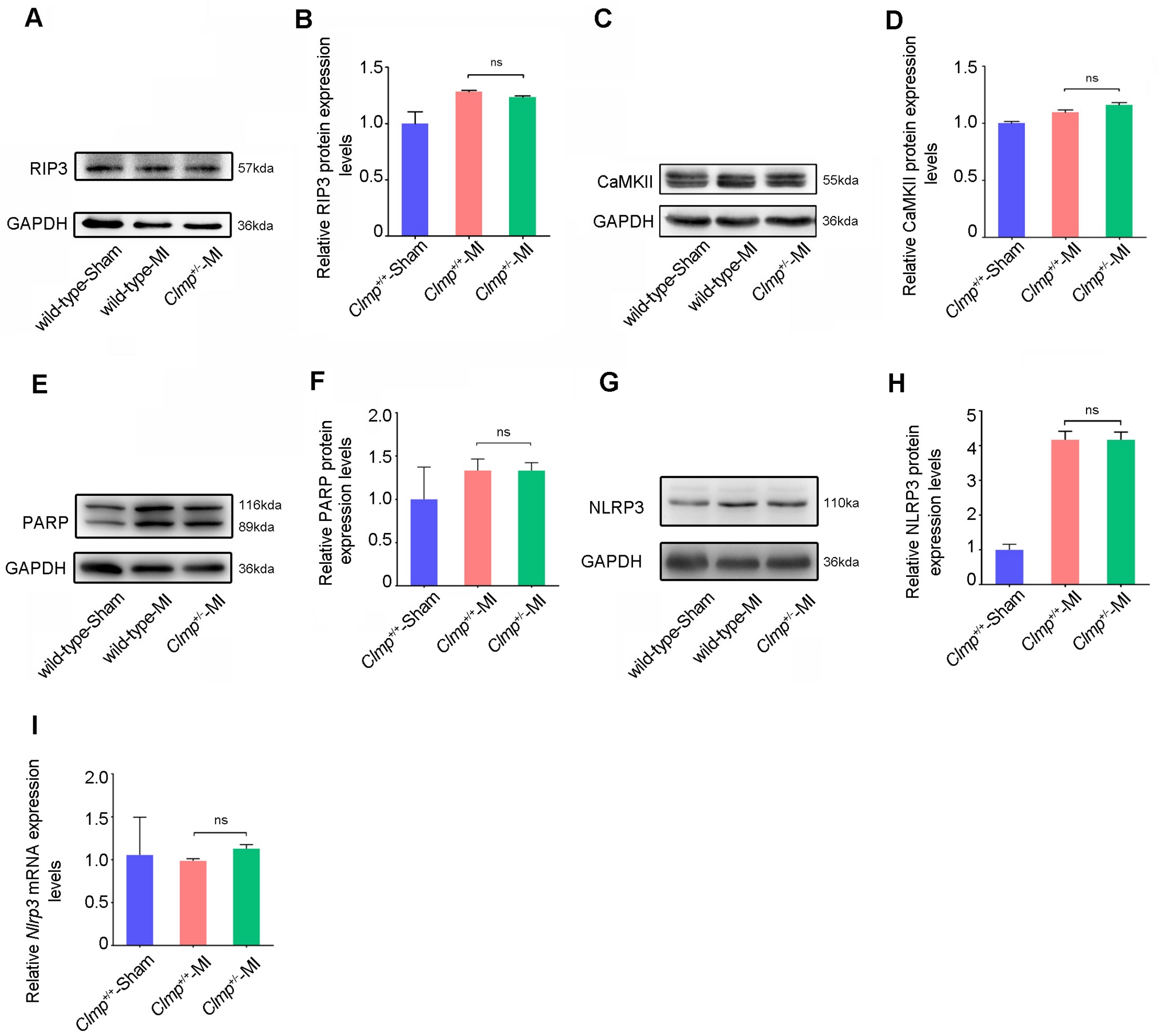

Supplemental Figure 5. Western blot analysis of cell necrosis markers in left ventricles of the wild-type shame, wild-type MI and *Clmp*$^{+/-}$ MI mice. (A) The presentative imaging of RIP3 protein expression in mouse LVs on day 3 post-MI. **(B)** The relative densitometric quantification of RIP3 protein on day 3 post-MI in panel A. **(C)** The presentative imaging of CaMKII protein expression in mouse LVs on day 3 post-MI. **(D)** The relative densitometric quantification of CaMKII protein on day 3 post-MI in panel C. (**E**) The presentative imaging of PARP protein expression in mouse LVs on day 3 post-MI. (**F**) The relative densitometric quantification of PARP protein on day 3 post-MI in panel E. (**G**) The presentative imaging of NLRP3 protein expression in mouse LVs on day 3 post-MI. (**H**) The relative densitometric quantification of NLRP3 protein on day 3 post-MI in panel H. (**I**) The relative mRNA expression of NLRP3 on day 3 post-MI.

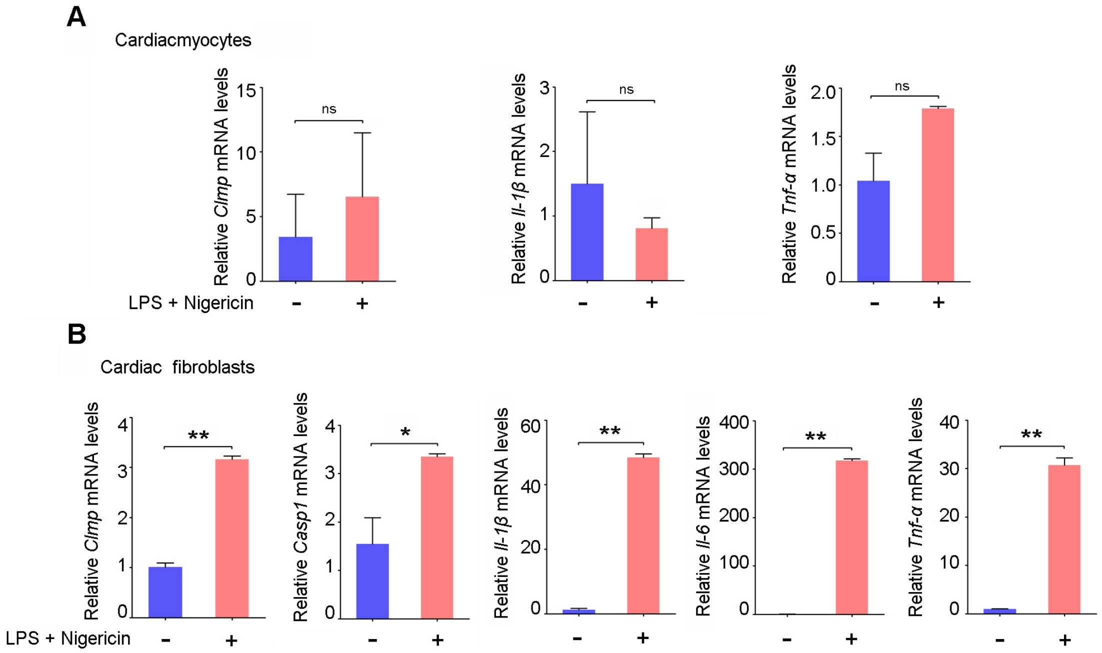

Supplemental Figure 6. Pyroptotic and inflammatory induction in cardiomyocytes and cardiac fibroblasts by LPS plus nigericin treatment. The qRT-PCR detection of *Clmp*, the pyroptosis-related gene *Casp1*, and inflammatory genes in cardiac myocytes (**A**) and cardiac fibroblasts (**B**), respectively. *$p<0.05$; **$p<0.01$; ns, not significant.

Supplemental Table 1. Primer sequences for quantitative PCR

Gene name	Primers
Clmp	F-GGAGAAGATGAACACCTGCC
	R-ACAGTCACCCGTACCACACA
C4b	F-GTGGTTCCTGCTCCCCAAAG
	R- CCACAGCTCCTCACATCTTCC
Ighm	F-CAGATCCGGTGACCATCGAG
	R-AGAAGGTGAGACCCCTGTGA
Igkc	F-CTTCCCACCATCCAGTGAGC
	R-AGTCCAACTGTTCAGGACGC
Ighg1	F-AACAGCACTTTCCGCTCAGT
	R-AAAGCTGCACTGTTGACCCT
Ccl8	F-GCTGTGGTTTTCCAGACCAA
	R-GAAGGTTCAAGGCTGCAGAA
IL-1β	F-TTGAAGTTGACGGACCCCAA
	R-GCTCTTGTTGATGTGCTGCT
IL-6	F-TCTTCAACCAAGAGATAAGCTGGA
	R-CGCACTAGGTTTGCCGAGTA
Tnf-α	F-CGGGCAGGTCTACTTTGGAG
	R-ACCCTGAGCCATAATCCCCT
J chain	F-AGGATCATCCCTTCCACCGA
	R-CCACTTCCACAGGATCGCAT
Gsdmd	F-CGCAGCCCCTATCTTCTACC
	R-GACGTGCTTCACCAACTCCA
Casp1	F-GCCCTGTTGGAAAGGAACTAAC
	R-AAATTGCTTCCTCTTTGCCCTC
Ly6G	F-AGAGGAAGTTTTATCTGTGCAGCC
	R-TCAGGTGGGACCCCAATACA
Nlrp3	F-ATCAACAGGCGAGACCTCTG
	R-GTCCTCCTGGCATACCATAGA
Anp	F- GATTGGAGCCCAGAGTGGAC
	R- CAGTGGCAATGTGACCAAGC
Bnp	F-CGGATCCGTCAGTCGTTTGG
	R-CACTTCAAAGGTGGTCCCAGA
18s	F-CTTAGAGGGACAAGTGGCG
	R-ACGCTGAGCCAGTCAGTGTA

Supplemental Table 2. All the antibodies for Western blot and Immunofluorescence

NAME	CAT. NO	MANUFACTURER	COUNTRY
CLMP	af7356	R&D	USA
GSDMD	sc-393656	Santa Cruz	USA
RIP3	17563-1-ap	Proteintech	CHINA
CaMKII	pa522168	Thermo	USA
caspase-1	pa522168	Adipogen	USA
PARP	9532	CST	USA
NLRP3	ab214185	Abcam	USA
p-histone H3	sc-8656-R	Santa Cruz	USA
histone H3	ab1791	Abcam	USA
p62	18420-1-AP	Proteintech	CHINA
IL-1β	ab9722	Abcam	USA
GAPDH	dkm9002	Sungene	CHINA
Cardiac Troponin T	15513-1-AP	Proteintech	CHINA
HRP AffiniPure Donkey Anti-Sheep IgG	A21060	Abbkine	CHINA
Peroxidase AffiniPure Goat Anti-Mouse IgG	115-035-003	Jackson ImmunoResearch	USA
Peroxidase AffiniPure Goat Anti-Rabbit IgG	111-035-003	Jackson ImmunoResearch	USA
Peroxidase AffiniPure Rabbit Anti-Goat IgG	305-035-003	Jackson ImmunoResearch	USA
Alexa Fluor® 594 AffiniPure Donkey Anti-Mouse IgG	715-585-151	Jackson ImmunoResearch	USA
Alexa Fluor® 488 AffiniPure Donkey Anti-Rabbit IgG	711-545-152	Jackson ImmunoResearch	USA

SUPPLEMENTAL REFERENCE

1. **Xiang FL, Fang M, Yutzey KE.** Loss of beta-catenin in resident cardiac fibroblasts attenuates fibrosis induced by pressure overload in mice. *Nat Commun*. 2017; 8: 712.
2. **McCarroll CS, He W, Foote K, Bradley A, McGlynn K, Vidler F, Nixon C, Nather K, Fattah C, Riddell A, Bowman P, Elliott EB, Bell M, Hawksby C, MacKenzie SM, Morrison LJ, Terry A, Blyth K, Smith GL, McBride MW, Kubin T, Braun T, Nicklin SA, Cameron ER, Loughrey CM.** Runx1 Deficiency Protects Against Adverse Cardiac Remodeling After Myocardial Infarction. *Circulation*. 2018; 137: 57-70.
3. **Kotter S, Kazmierowska M, Andresen C, Bottermann K, Grandoch M, Gorressen S, Heinen A, Moll JM, Scheller J, Godecke A, Fischer JW, Schmitt JP, Kruger M.** Titin-Based Cardiac Myocyte Stiffening Contributes to Early Adaptive Ventricular Remodeling After Myocardial Infarction. *Circ Res*. 2016; 119: 1017-29.
4. **W C Claycomb NALJ, B S Stallworth, D B Egeland, J B Delcarpio, A Bahinski, N J Izzo Jr.** HL-1 Cells: A Cardiac Muscle Cell Line That Contracts and Retains Phenotypic Characteristics of the Adult Cardiomyocyte. *Proc Natl Acad Sci U S A*. 1998; 95: 6.
